# C-Phycocyanin prevents acute myocardial infarction-induced oxidative stress, inflammation and cardiac damage

**DOI:** 10.1080/13880209.2022.2055089

**Published:** 2022-04-02

**Authors:** Vanessa Blas-Valdivia, Daniela Nikita Moran-Dorantes, Placido Rojas-Franco, Margarita Franco-Colin, Neda Mirhosseini, Reza Davarnejad, Ahmad Halajisani, Omid Tavakoli, Edgar Cano-Europa

**Affiliations:** a Departamento de Fisiología, Escuela Nacional de Ciencias Biológicas, Laboratorio de Neurobiología, Instituto Politécnico Nacional, Ciudad de México, Mexico; b Departamento de Fisiología, Escuela Nacional de Ciencias Biológicas, Laboratorio de Metabolismo I, Instituto Politécnico Nacional, Ciudad de México, Mexico; c Chemical Engineering Department, Engineering Faculty, Arak University, Arak, Iran; d Biofuel Laboratory, Caspian Faculty of Engineering, College of Engineering, University of Tehran, Tehran, Iran; e School of Chemical Engineering, College of Engineering, University of Tehran, Tehran, Iran

**Keywords:** Phycobiliproteins, cardioprotection, anti-inflammatory, heart damage, cardiotoxicity

## Abstract

**Context:**

C-Phycocyanin is a protein with anti-scavenger, antioxidant and anti-inflammatory actions against agents that cause cellular damage. The cardioprotective action of C-phycocyanin against acute myocardial infarction (AMI) has not been studied in animal models.

**Objective:**

To investigate C-phycocyanin’s effect on oxidative stress, inflammation and cardiac damage in a model of isoproterenol-induced AMI.

**Materials and methods:**

Wistar rats were divided into four groups: (1) sham + vehicle (0.9% saline solution by oral gavage, OG); (2) sham + C-phycocyanin (50 mg/kg/d, OG); (3) AMI + vehicle, and (4) AMI + C-phycocyanin. AMI was induced by administering isoproterenol (20, 10, 5 and 3 mg/kg each dose per day), and serum cardiac enzymes were quantified. After five days, the animals were euthanized; the heart was dissected to determine oxidative stress, redox environment, inflammation and cardiac damage markers.

**Results:**

We observed that C-phycocyanin reduced AMI-increased cardiac enzymes (CK by about 53%, CKMB by about 60%, AST by about 16% and ALT by about 21%), lipid peroxidation (57%), reactive oxygen species (50%), nitrites (46%), oxidized glutathione (41%), IL1β (3%), INFγ (5%), TNFα 3%), Bcl2 (37%), Bax (43%), COX2 (21%) and caspase 9 (61%). Finally, C-phycocyanin reduced AMI-induced aberrant histological changes related to myonecrosis, interstitial oedema and inflammatory infiltration in the heart muscle.

**Conclusions:**

C-Phycocyanin prevents AMI-induced oxidative stress, inflammation and heart damage. This study is the first report that employed C-phycocyanin in an animal model of AMI and supports the potential use of C-phycocyanin in the management of AMI.

## Introduction

Acute myocardial infarction (AMI) is a disease that causes myocardial cell death due to prolonged ischaemia. It is the most severe manifestation of coronary artery disease that affects about 7 million individuals worldwide each year (Thygesen et al. 2012). AMI is a worldwide public health problem that causes more than 2.4 million deaths in the USA, more than 4 million deaths in Europe and northern Asia, and more than a third of deaths in developed nations (Nichols et al. 2014). This disease has a significant economic impact because it has been reported that about 1.1 million hospitalizations with an estimated direct cost of at least US$450 billion (Benjamin et al. 2019). There are some reports about one death every 4.3 min in Mexico for ischaemic heart disease (Mendoza-Herrera et al. 2019). The patients who received timely treatment had more probability of surviving, but their long-term complications are the first subsequent medical consultation; this represents a running cost of about US$16.6 million (Reed et al. 2017). Coronary artery disease is responsible for about 46% of mortality of reported cases in Iran. It annually leads to approximately 6.3 million admissions to the hospitals affiliated with the Iranian Ministry of Health and Medical Education (Sharif Nia et al. 2018). The ischaemic heart disease has become the leading contributor to disease burden as assessed based on disability-adjusted life-years (Borrayo-Sánchez et al. 2018).

The pathophysiology of AMI involves myocardial ischaemia that promotes an imbalance between oxygen supply and demand. This causes disturbance in the redox environment. Furthermore, it promotes a rise in oxidative stress and cellular damage. The factors influencing the severity of ischaemia include whether the vessel was wholly or partially occluded, during occlusion, amount of myocardium supplied, presence of collateral vessels, as well as reperfusion’s adequacy following treatment. Thus, timely treatment is an essential issue to avoid patient death or long-term complications. Percutaneous coronary intervention (PCI) and thrombolysis are the most specific treatment as part of the revascularization processes; although, time is one of the limiting factors in patients suitable for that treatment (for PCI, the door-to-balloon time must be less than 90 min). Meanwhile, the door-to-needle time must be less than 30 min (Thygesen et al. 2012; Reed et al. 2017).

Therefore, it is necessary to develop new treatments to allow more time for revascularization procedures. The C-phycocyanin as a nutraceutical has a potential coadjutant role in the AMI treatment. C-Phycocyanin is a phycobiliprotein in cyanobacteria, which can assist the photosynthesis process. It has a deep and intense blue colour due to forming alpha (α) and beta (β) protein subunits with isomeric linear tetrapyrrole prosthetic groups (phycocyanobilin chromophore). C-Phycocyanin has been used as an antioxidant and anti-inflammatory treatment for its structure and pharmacological security, avoiding oxidative stress and cell damage (Romay et al. 2003). It has been reported as neuroprotective (Rimbau et al. 1999; Romay et al. 2003), nephroprotective (Rodríguez-Sánchez et al. 2012; Memije-Lazaro et al. 2018; Rojas-Franco et al. 2018, 2021), hepatoprotective (Sathyasaikumar et al. 2007; Ou et al. 2010), as well in fact the C-phycocyanin prevents oxidative stress and cell damage *in vitro* as the hypoxia model employing myoblast cell line H9c2 (Gao et al. 2019) or doxorubicin-induced cardiotoxicity in adult ventricular cardiomyocyte culture (Khan, Varadharaj, Ganesan, et al. 2006). Moreover, an *in vivo* model of ischaemia/reperfusion with isolated rat hearts has also been studied (Khan, Varadharaj, Shobha, et al. 2006). One of the recent model advantages is the C-phycocyanin amount measurement while the major disadvantage is administration manner and the C-phycocyanin metabolism in the whole of organism physiological responses.

In this study, the cardioprotective activity of C-phycocyanin against AMI caused by isoproterenol on oxidative stress, inflammation and cardiac damage markers was studied.

## Materials and methods

### Animals

Thirty male Wistar rats 3-months-old with weight of 250–300 g were used in this study. They were housed in groups of 5 and 6 in Plexiglas cages with food and water *ad libitum*. The housed room had a regulated temperature (21 ± 2 °C), and a 12 h light/dark cycle (light on at 8:00 am). All procedures applied in this research were according to the Mexican Laws and Codes’ guidelines as issued in the Mexican Official Standard NOM-062-ZOO-1999 for the production, care and use of laboratory animals. Internal Bioethics Committee (CEI) approved this protocol with the number approbation CEI-ENCB ZOO-017-2018.

The animals were randomly assigned to four groups: (1) sham + vehicle (0.9% saline solution by oral gavage, OG); (2) sham + C-phycocyanin (50 mg/kg/d, OG); (3) AMI + vehicle and (4) AMI + C-phycocyanin. The sham groups had six animals while the AMI groups had nine animals.

C-Phycocyanin was purified from *Arthrospira maxima* (Oscillatoriaceae) (Setchell and Gardner 1917) cultured in our laboratory in Zarrouk medium (Memije-Lazaro et al. 2018; Rojas-Franco et al. 2021). A Sephadex G-25 column was equilibrated with 100 mM phosphate buffer at pH of 7.4. The exclusion chromatography was eluted with a pH 7.4 phosphate buffer with a linear gradient from 100 mM to 6.5 mM. The protein was precipitated with (NH_4_)_2_SO_4_ at 4 °C and dialysed. The fraction was passed through ion-exchange chromatography using DEAE-cellulose equilibrated with 50 mM acetate buffer pH 5.5. The column was eluted with a 50 mM acetate buffer (pH 5.5). Finally, the protein was precipitated with (NH_4_)_2_SO_4_ at 4 °C, dialysed, and lyophilized. The extracted C-phycocyanin had a purity index of 4.1 (*A*_620_/*A*_280_=4.1).

AMI was induced by the administration of isoproterenol (I5627, Sigma-Aldrich, Darmstadt, Germany) in a scheme of four doses in four days (20, 10, 5 and 3 mg/kg each dose was subcutaneously administrated daily at 9:00 am) (Geng et al. 2004). Meanwhile, the treatment with C-phycocyanin was administrated 30 min after isoproterenol administration.

The cardiac enzymes as total creatine kinase (CK) and the isoform MB-CK, as well as alanine aminotransferase (ALT) and aspartate aminotransferase (AST) from a blood sample (200 µL) at 6, 24, 48 and 72 h after the first doses of isoproterenol were determined. The enzymatic activity was measured according to the manufacturer’s instruction (RANDOX, Estado de Mexico SA. de CV., México), and the absorbencies were measured with a UV-visible spectrophotometer. After five days, the animals were euthanized with sodium pentobarbital (35 mg/kg intraperitoneally). The heart was dissected, and the transversal section was frozen for the determination of the oxidative stress, redox environment, expression of proteins involved in cardiac damage, and the interleukin mRNA synthesis using a RT-PCR method.

### Oxidative stress and redox environment markers

The cardiac sections were homogenized in 3 mL of 10 mM phosphate buffer (pH 7.4) to be used for all biochemical tests (Memije-Lazaro et al. 2018; Rojas-Franco et al. 2019). The oxidative stress and redox environment markers evaluated were lipid peroxidation, reactive oxygen species (ROS), nitrites, and reduced and oxidized glutathione (GSH and GSSG, respectively).

Lipid peroxidation was quantified using the technique proposed by Memije-Lazaro et al. (2018) and Rojas-Franco et al. (2019). An aliquot (500 µL) of homogenized cardiac section was added to 4 mL chloroform:methanol (2:1, v/v) and agitated. The solution was left to separate in aqueous and chloroform phase at 4 °C for 30 min in dark. The aqueous phase was discarded and 2 mL of organic phase. Fluorescence was determined using an RF5000U Shimadzu Spectrophotometer (Kyoto, Japan) at 370 nm (excitation) and 430 nm (emission) wavelengths. The results were expressed as relative fluorescence units (RFU) per milligram of protein.

The ROS were measured by the formation of 2,7-dichlorofluorescein (DCF). The homogenate (10 µL) was added to 1945 µL of 40 mM TRIS–10 mM HEPES (18:1 v/v) and incubated in the presence of 50 µL of 2,7-dichlorofluorescin diacetate (DCFH-DA) for 1 h at 37 °C. The reaction was stopped by freezing (Rodríguez-Sánchez et al. 2012). The fluorescence was measured in the spectrophotometer at 488 nm (excitation) and 525 nm (emission) wavelengths. The results were expressed as ng of DCF formed/mg protein/h (Memije-Lazaro et al. 2018; Rojas-Franco et al. 2019).

Nitrites were measured as indirect markers of nitrergic stress marker. Five hundred microlitres of homogenate was added to 500 µL concentrated hydrochloric acid and 500 µL of 20% of zinc suspension. The mixture was stirred and incubated at 37 °C for 1 h, followed by centrifugation at 4000 rpm for 2 min. Supernatant (50 µL) was added in 96-well polystyrene plates containing 50 µL of 0.6% sulphanilamide and 0.12% *N*-(naphthyl)-ethylenediamine and incubated for 15 min at room temperature. The absorbance was measured at 530 nm in a Multiscan Go^®^ plate spectrophotometer (Memije-Lazaro et al. 2018; Rojas-Franco et al. 2019).

REDOX environment markers (GSH and GSSG) were measured with a sample containing 300 µL treated with 30% phosphoric acid and centrifuged at 10,000×*g* for 30 min at 4 °C. To determine GSH, 30 µL of the supernatant diluted 1:10 with FEDTA (100 mM phosphate and 5 mM EDTA) was added to 1.9 mL of FEDTA. The mixture was reacted with 100 µL of o-phthaldialdehyde. To measure the GSSG, 130 µL of supernatant was added to 60 µL of *N*-ethylmaleimide for 30 min then, mixed with 1.84 mL of FEDTA, and 100 µL of o-phthaldialdehyde for the post-incubation. The two chemical species were measured through the spectrophotometer (*γ*_excitation_=350 nm and *γ*_emission_=420 nm). The results were expressed as ng of GSH or GSSG/mg protein, and the GSH^2^/GSSG ratio was used as an indicator of the REDOX environment (Memije-Lazaro et al. 2018; Rojas-Franco et al. 2019).

### Expression of proteins involved in the cellular death and inflammation

The cardiac section was homogenized in 3 mL of 10 mM phosphate buffer (pH 7.4) containing UltraCruz^®^ protease inhibitor cocktail. Protein sample (50 µg) was charged in 10% polyacrylamide gels and they were separated by vertical electrophoresis (120 mV for 120 min). Then, the sample was electrotransferred to PVDF membranes using a Trans-Blot Turbo Transfer System (Bio-Rad, Hercules, CA) for 9 min (25 V, 2.5 A). After that, the membranes blocked for 1 h in blocking buffer (PBST; 0.05% Tween 20 in saline phosphate buffer containing 5% low-fat milk Svelty^®^) under a constant agitation. After blocking, membranes were incubated at 4 °C overnight with the primary antibodies (Santa Cruz Biotechnology, Dallas, TX) diluted 1:1000 for Bax (sc-20067), BCl2 (sc7382) p-NFkB p65 (sc-101752), COX2 (sc-23983) and diluted 1:500 for NOS2 (sc-7271). After incubation, membranes were washed three times with fresh PBST (20 min per wash) and then incubated in 1:2000 diluted secondary antibody (HPR-conjugated from Life Technologies, Rockford, IL) for one 1 h at room temperature, under constant stirring. Then, membranes were washed three times with fresh PBST and protein bands revealed in photographic plates (JUAMA, Mexico) by chemiluminescence, using Luminata™ Forte^®^ (Millipore, Billerica, MA). Protein β-actin expression was used as charge control and constitutive protein (Santa Cruz Biotechnology, Dallas, TX; sc-1615, dilution: 1:4000). The optical density (O.D.) from all obtained bands was analysed by the Image J program (NIH, Bethesda, MD; version 1.51p).

For the RT-PCR, total RNA of heart myocardial cells was extracted and performed with 200 µL of TRIzol reagent (Invitrogen™, Carlsbad, CA) following the manufacturer’s instructions. The total RNA concentration was quantified in iDrop from Multiskan Go (Thermo Scientific, Co., Waltham, MA). The iScript^TM^ select cDNA synthesis kit (Bio-Rad, Hercules, CA, cat. 170-8897) was used for the retrotranscription process following the manufacturer’s instructions. The reactions were performed at 42 °C for 1 h. Then, the reverse transcriptase was inactivated with the incubation at 85 °C for 5 min. All cDNA samples were stored at −20 °C. The cytokines gene expression was evaluated with polymerase chain reaction (PCR) using the OnePCR^TM^ Ultra kit (GeneDireX, MB208-0100). All reactions were carried out in 4 µL of OnePCR^TM^ mix, 1 µL of 10 µM forward primer, 1 µL of 10 µM reverse primer, 1 µL of cDNA, and 1 µL of sterilized water. All genes were amplified with the following settings: 30 s of denaturation at 95 °C, 30 s of annealing as indicated in the Supplementary data (S1) and 30 s of extension at 72 °C for 35 cycles and a final extension step at 72 °C for 5 min. PCR products (5 µL) were mixed with 2 µL of Novel Juice (GeneDireX, LD001-1000). The mixture was charged in 1% agarose gel and separated by electrophoresis (50 mV for 100 min). The gel was photo-documented, and the relative expression of cytokines genes was analysed by densitometry of gel bands and normalized to β-actin using Image J program version 1.51p (NIH, Bethesda, MD).

### Determination of caspase 9 activity

The activity of caspase 9 was assessed using a commercial colorimetric assay kit. Chemicon International (Temecula, CA, catalogue no. APT173). According to the manufacturer’s instructions, the caspase activity was detected by cleavage of *p*-nitroaniline (pNA) from Leu-Glu-His-Asp-*p*-nitroanilide (LEHD)-pNA (Rojas-Franco et al. 2018).

### Statistical analysis

All data are presented as mean ± standard error of the mean. The cardiac enzyme evaluation (AST, ALT, CK and CKMB) was analysed by three-way repeated-measures analysis of variance (RM-three-way ANOVA) and the Tukey *post hoc* test. The factors were according to the AMI, treatment with C-phycocyanin, and time. The factors were analysed with two-way ANOVA and Tukey’s *post hoc* test. These factors were according to the AMI and treatment with C-phycocyanin.

## Results

**Figure 1.**
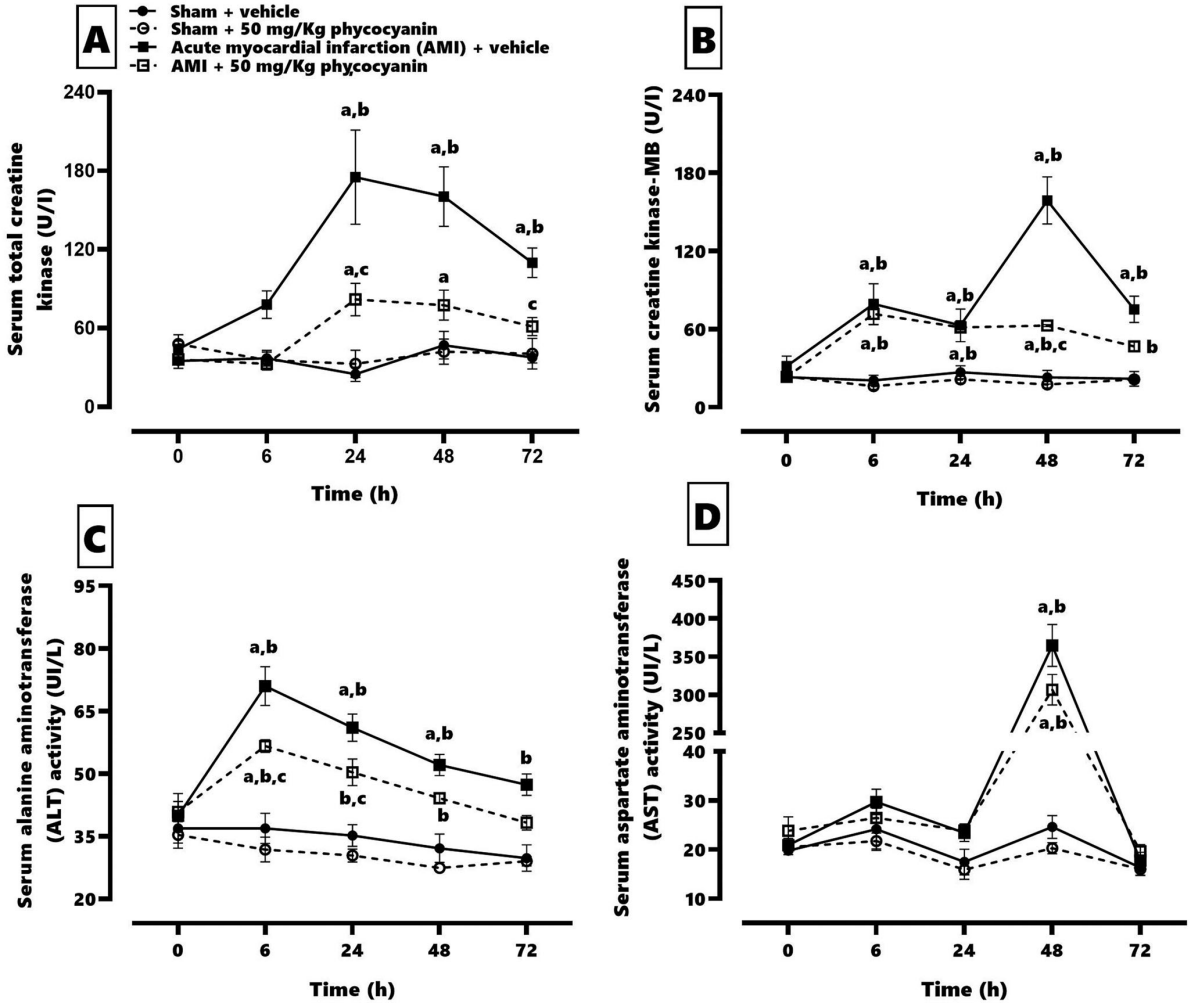
Effect of phycocyanin on serum cardiac enzymes related to acute myocardial infarction (AMI). The figure shows the quantification of serum total creatinine (A), serum creatinine kinase-MB (B), serum alanine aminotransferase (C) and serum aspartate aminotransferase (D). Data represent the mean ± SEM. Three-way repeated measures ANOVA and Tukey post hoc. (a) *p* < 0.05 compared with t = 0; (b) *p* < 0.05 compared with the AMI group; (c) *p* < 0.05 compared with its respective vehicle group.

Figure 1 shows the effect of C-phycocyanin on serum cardiac elevation caused by AMI. AMI enhanced the CK (panel A) from 24 h to 72 h after isoproterenol administration (24 h = 546%, 48 h = 382% and 72 h = 268%) with an elevation of the specific isoform MB (panel B) at 48 and 72 h (24 h 234%, 48 h = 687% and 72 h = 357%). AMI increased the ALT (panel C) serum levels since 6 h after isoproterenol injection (6 h = 190%, 24 h = 173%, 48 h = 162% and 72 h = 159%). The AST (panel D) serum levels increase at 48 h by about 1480% after isoproterenol administration. Meanwhile, the C-phycocyanin treatment prevented the CK elevation (24 h = 249%, 48 h = 184%), and it reduced the CKMB elevation by about 60% at 48 h. It also restored the normal CKMB levels at 72 h after isoproterenol administration. However, C-phycocyanin could not modify the serum AST elevation, but it could reduce 18–25% of the ALT serum levels concerning the AMI group during the evaluation time.

**Figure 2.**
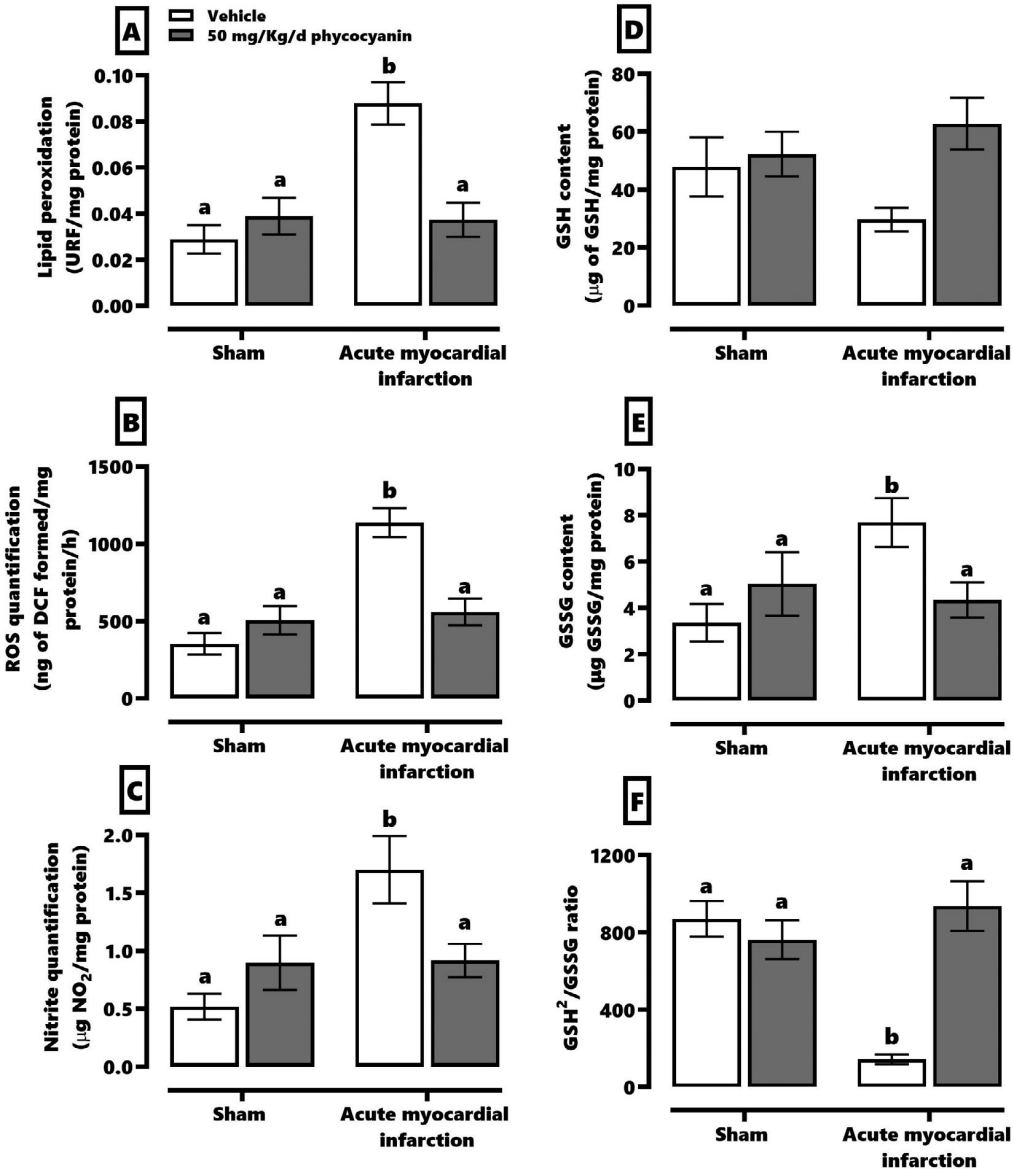
Effect of phycocyanin on acute myocardial infarction (AMI)-causes oxidative stress and redox markers disturbance in the heart. The figure shows the quantification of lipid peroxidation (A), ROS (B), and nitrites (C) as oxidative stress markers. Meanwhile, the redox environment markers evaluated were GSH (D), GSSG (E), and GSH^2^/GSSG ratio (F). Data represent the mean ± SEM. Two-way ANOVA and Tukey post hoc. a ≠ b *p* < 0.05.

Figure 2 shows the effect of C-phycocyanin on oxidative stress and redox environment markers. AMI causes an increase of threefold in lipid peroxidation (panel A), ROS (panel B) and nitrites (panel C). According to the redox environment, AMI increased about 40% of GSSG (panel E), reduces about 37% GSH (panel D) and 86% of the GSH^2^/GSSG ratio (panel F). The treatment with C-phycocyanin prevented elevation of AMI-induced oxidative stress markers.

**Figure 3.**
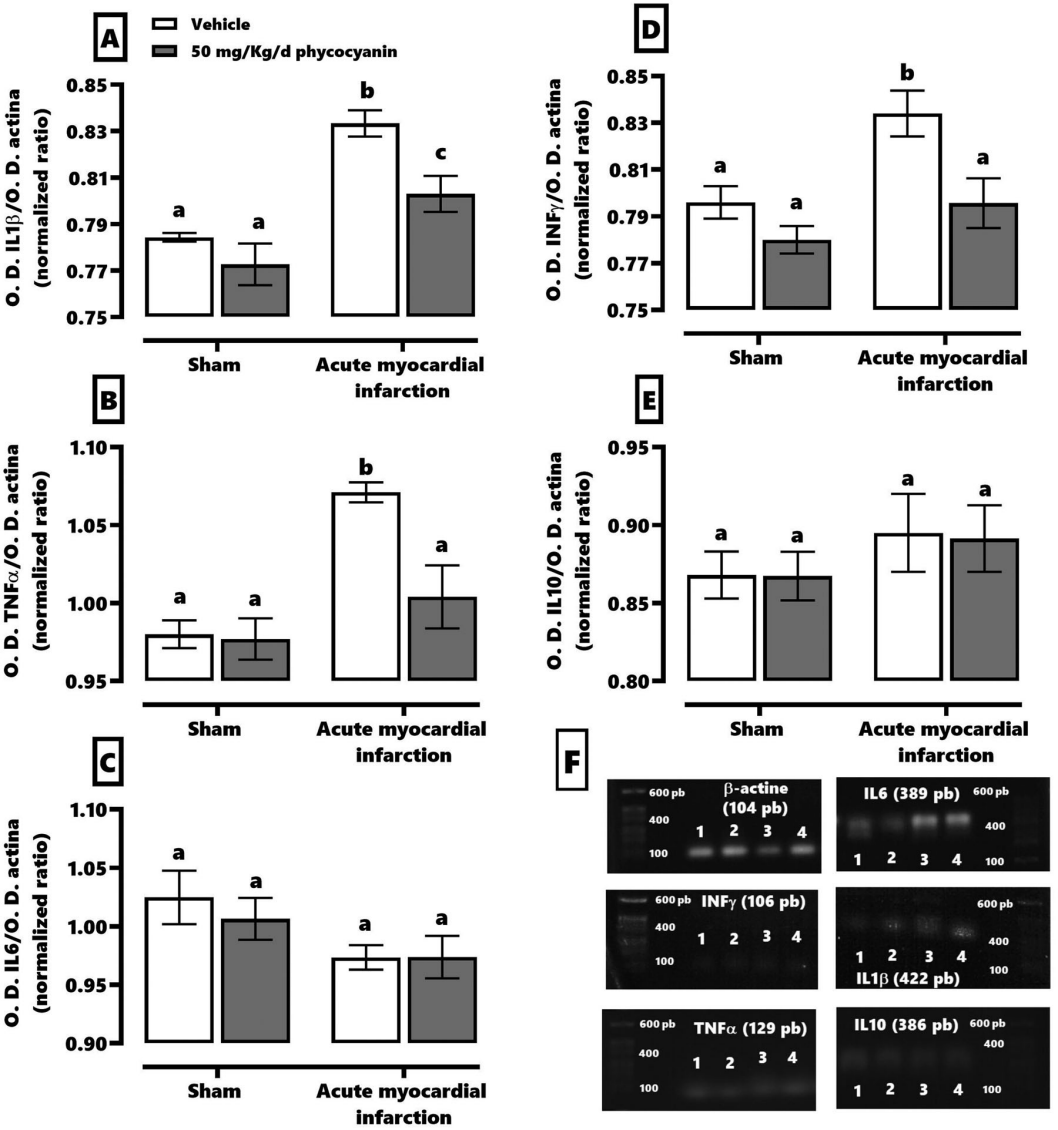
Effect of phycocyanin on acute myocardial infarction (AMI)-causes inflammation in the heart. The figure shows the quantification of the synthesis of mRNA of IL1β, (A), TNFα (B), IL6 (C), INFγ (D), and IL10 (E). Panel F shows the blot of the amplicon of the RT-PCR technique of each group: vehicle + SS [1], sham + phycocyanin [2], IAM + SS [3], IAM + phycocyanin [4]. Data represent the mean ± SEM. Two-way ANOVA and Tukey post hoc. a ≠ b ≠ c *p* < 0.05).

Figure 3 demonstrates that C-phycocyanin prevented AMI-induced inflammation. It showed that AMI increases around 10–15% the synthesis of mRNA for IL1β (panel A), TNFα (panel B) and INFγ (panel D) without a change in the synthesis of IL6 (panel C) and IL10 (panel E). The C-phycocyanin treatment normalized TNFα and INFγ mRNA synthesis levels with a mild increase of IL1β about 3%.

**Figure 4.**
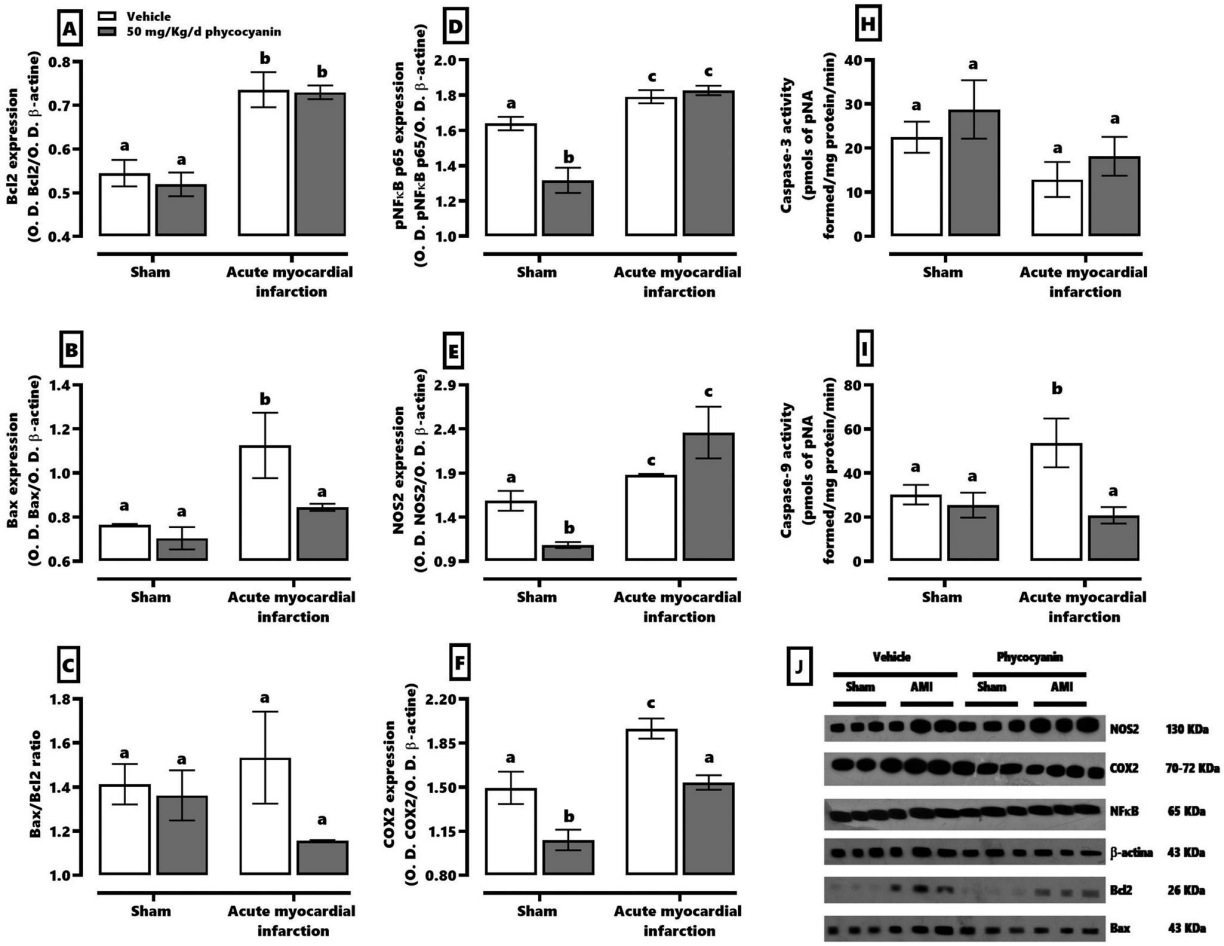
Effect of phycocyanin on acute myocardial infarction (AMI) causes over-expression of proteins involved in the heart’s inflammatory and cell death process. The figure shows the expression of Bcl2 (A), Bax (B), Bax/Bcl2 ratio (C), phospho-NFkB p65(D), NOS2 (E), and COX2(F), as well as the activities of caspase 3 (H) and caspase 9 (I). Panel J shows the expresión blot of all proteins by western blotting technique. Data represent the mean ± SEM. Two-way ANOVA and Tukey post hoc. a ≠ b ≠ c *p* < 0.05.

The expression of proteins related to inflammation and cell damage can be seen in Figure 4. This shows that AMI increased Bcl2 (panel A) and Bax (panel B) about 37 and 43%, respectively. This pathology augmented the inflammatory protein expression about 12–30% and the activity of effector caspase 9 (panel I), as well. On the other hand, the sham group with C-phycocyanin treatment reduced the expression of phospho-NFκB p65 (20%, panel D), NOS2 (32%, panel E) and COX2 (19%, panel F). Moreover, the C-phycocyanin treatment for AMI only restores the Bax expression and increases caspase 9 activity.

**Figure 5.**
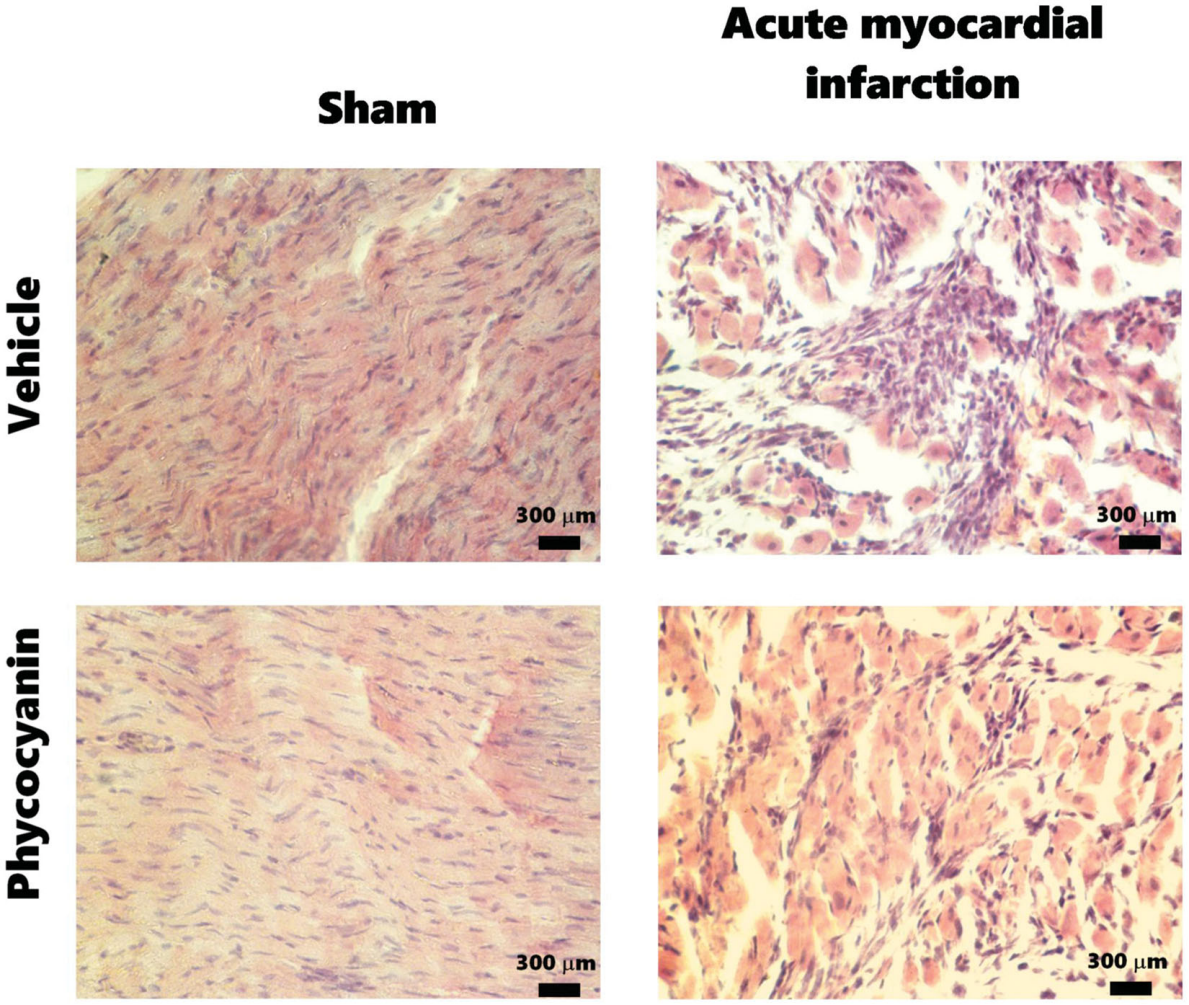
Effect of phycocyanin on acute myocardial infarction (AMI)-causes heart damage. The photomicrographs show a topographic image of the myocardium. Sham groups had a longitudinal myocardium layer that contains cross-striated muscle cells (cardiomyocytes) with one centrally placed nucleus. The cardiomyocytes are arranged in spirals or longitudinally attached to the cardiac skeleton can be dense connective tissue. The AMI photomicrographs show that this pathology promotes edema with massive necrosis of cardiac muscle fibers with different vacuolar change grades and inflammatory infiltrated zones. However, phycocyanin treatment reduced the edema, necrosis, and inflammatory process. Technique stain is hematoxylin & eosin.

Figure 5 shows the effect of C-phycocyanin on AMI-induced heart damage. The photomicrographs of sham groups show evident integrity of the longitudinal myocardial cell membrane near to epicardium. The myocardium contains cross-striated muscle cells (cardiomyocytes) with one centrally placed nucleus. The cardiomyocytes are arranged in spirals or longitudinally attached to the cardiac skeleton, which may make connective tissue. The AMI photomicrographs show that this pathology promotes interstitial oedema with massive necrosis of cardiac muscle fibres with different vacuolar change grades and inflammatory infiltrated zones; although, the C-phycocyanin treatment reduces the oedema, necrosis and inflammatory process.

## Discussion

Isoproterenol, a β-agonist drug, can reproduce severe stress in the myocardium resulting in infarct-like with necrosis in the heart. This model may increase serum cardiac enzymes, oxidative stress, inflammation and myocardial cells death (Devika and Stanely Mainzen Prince 2008; Jia et al. 2017). Isoproterenol is a synthetic catecholamine which would increase heart frequency, blood pressure and vascular resistance (Nikam et al. 2008). These physiological events increase myocardial oxygen demand and reduce the myocardial oxygen supply due to aerobic metabolism in the cardiac muscle. Hence, the myocardium is susceptible to ischaemia. The ischaemia-induced intramyocardial ATP depletion modifies the sodium and calcium currents, consequent contractile arrest and cellular swelling. The cardiomyocyte’s death releases the cell content, and cardiac enzymes elevate in the extracellular compartment (Tibaut et al. 2017). Furthermore, isoproterenol’s metabolism in mammals produces free radicals when adrenochrome–adrenolutin dimer is produced as a final metabolite (Rupp et al. 1994). Thus, the isoproterenol disturbs the redox environment that promotes interstitial oedema with massive necrosis of cardiac muscle fibres (Rathore et al. 1998). Moreover, the inflammatory process is activated in the infarcted heart to release pro-inflammatory cytokines such as TNFα, IL1β and IL-6 when necrosis occurs. The inflammatory cytokine upregulation happens due to increasing ROS and phospho-NFκB p65. The last regulatory protein also upregulates the transactivation of target genes related to the cellular proliferation, inflammation and apoptosis (Neri et al. 2015). The animals infarcted with isoproterenol reproduce all of the mentioned physiological processes, although the C-phycocyanin treatment prevents AMI-induced cardiac damage by lowering oxidative stress and inflammation. This disturbs the redox environment and cells death with increasing the myocardial enzymes of serum. Some mechanisms which explain the nutraceutical effect as the antioxidant, anti-inflammatory and cell protection action have been reported in the literature (Romay et al. 2003; Khan, Varadharaj, Shobha, et al. 2006).

In fact, C-phycocyanin (as a phycobiliprotein with a molecular weight of 112 kDa; Patel et al. 2005) is a nucleophilic compound which can directly interact with the oxidants (Romay et al. 2003). It covalently tends to link to the open-chains of tetrapyrrole moieties and phycocyanobilin (Hirata et al. 2000). Hence, C-phycocyanin acts as a prodrug due to its digesting by gastrointestinal enzymes and breaking chromo-peptides (Minic et al. 2016). In the serum, phycocyanobilin binds to albumin (due to its low solubility in water), extending its therapeutical effects into the whole of organs (Radibratovic et al. 2016). Similar research with positive results has been conducted with acute kidney injury (Rodríguez-Sánchez et al. 2012; Garcia-Pliego et al. 2021; Rojas-Franco et al. 2021).

Moreover, C-phycocyanin prevents the redox environment’s disturbance and inactivates signalling pathways during the inflammation process. In fact, ROS enhances phospho-NFκB p65 and increases the mRNA synthesis during the inflammatory cytokines and apoptosis promoters. A similar result has been found by Hao et al. (2018). They prevented the inflammation process by phospho-NFκB p65 and PCD5 (programmed cell death 5) because C-phycocyanin has a positive effect against AMI causing over phospho-NFKB p65 and mRNA synthesis for IL1β, TNFα and INFγ.

Heme oxygenase-1 (HO-1) is another molecular pathway activated by C-phycocyanin related to antioxidant and anti-inflammatory processes. In fact, HO-1 through PKC α/β II (protein kinase C)/Nrf-2 (nuclear factor erythroid-derived 2) promotes the caspase reduction and cell death (Kim et al. 2018) and antioxidant enzyme increment (Gao et al. 2016). Moreover, another signalling pathway that contributes to the caspase reduction is the endoplasmic reticulum stress decline through the inositol-requiring enzyme-1α (IRE-1α) due to C-phycocyanin treatment (Rojas-Franco et al. 2021). Rojas-Franco et al. (2018) found positive effects of C-phycocyanin for preventing the redox environment disturbance in the acute kidney injury as expected in the infarcted heart (according to the current research). On the other hand, C-phycocyanin could prevent AMI-induced endothelial dysfunction because *Spirulina* ethanol extract promotes the relaxing of aortic rings (Mascher et al. 2006). It seems that C-phycocyanin will also prevent endothelial dysfunction and promote the vasorelaxant of heart vessels avoiding ischaemia induced by AMI. However, this proposal must be analysed to describe the nutraceutical effect on endothelial function in future research. Also, it previously was described that C-phycocyanin is a prodrug. Thus, the limitation of the study is related to the C-phycocyanin metabolism and the metabolite(s) responsible(s) for the cardioprotective effect. We proposed that this protein is metabolized to phycocyanobilin, a responsible molecule of some nutraceutical action as the nephroprotective effect (Garcia-Pliego et al. 2021). However, the use of phycocyanobilin for AMI treatment in future research could explain the cardioprotective effect.

Finally, in the framework of integrated results, C-phycocyanin as AMI treatment can limit hypertensive response and the cardiotoxic effect of ischaemia, prolonging the door-to-balloon time (for thrombolysis), and door-to-needle time (for PCI) during AMI treatment.

## Conclusions

C-Phycocyanin had a cardioprotective nutraceutical effect with *in vitro* models and in the isolated organ system. However, the metabolism effect in the complete organism has not been previously described. This study is the first report that employed C-phycocyanin in an animal model of AMI. It prevents oxidative stress and inflammation because C-phycocyanin down-regulates iNOS, COX2 and phospho-NFκB p65, reducing the mRNA synthesis of IL1β and TNFα. Finally, C-phycocyanin can be used as a coadjuvant in AMI treatment to give more time to the heart physicians for definitive treatments such as thrombolysis and PCI.
